# Collapsing focal segmental glomerulosclerosis secondary to COVID-19: A systematic review and meta-analysis

**DOI:** 10.1097/MS9.0000000000000107

**Published:** 2023-02-07

**Authors:** Mohammad A. Qamar, Lucas M. Kogut, Sameer S. Tebha, Aabiya Arif, Jesse Ninmol, Muhammad R. Abdul Razzaque, Khulud Qamar, Abubakr Yosufi

**Affiliations:** a Ziauddin University; b Dow Medical College; c Department of Neurosurgery and Neurology, Jinnah Medical and Dental College; d Department of Medicine, Section of Nephrology, Jinnah Medical and Dental College, Karachi, Sindh, Pakistan; e Department of Public Health, University of California, Berkeley, California; f Department of Nephrology, Hope Medical Institute, Newport News, Virginia, USA; g Medical School, Kabul University of Medical Sciences, Kabul, Afghanistan

**Keywords:** collapsing glomerulopathy, COVID-19, focal segmental glomerulosclerosis, glomerulosclerosis, SARS-CoV-2

## Abstract

The renal system manifestations of coronavirus disease-2019 have been documented extensively; however, scientific literature remains scarce regarding collapsing glomerulopathy hence the need for this investigation.

**Methods:**

A comprehensive review was conducted covering a timeline from 1 January 2020 to 5 February 2022 without any restrictions. The data extraction was conducted independently, and articles were assessed for the risk of bias. Data analysis was conducted using Comprehensive Meta-Analysis version 3.3.070 and RevMan version 5.4 for pooled proportions and risk ratio (RR) between dialysis-dependent and independent treatment groups with a *P*-value less than 0.05 considered significant.

**Results:**

A total of 38 studies were included in this review, including 74 (65.9%) males. The mean age was 54.2 years old. The most common symptoms reported were related to the respiratory system (59.6%, 95% CI: 50.4–68.2%) and hematuria (34.2%, 95% CI: 26.1–43.4). Antibiotics (25.9%, 95% CI: 12.9–45.3%) was the commonest management used. Proteinuria was the most reported laboratory finding at 89.5% (95% CI: 82.4–93.9%), while the commonest microscopic finding was acute tubular injury (77.2%, 95% CI: 68.6–84.0%). An increased risk of the presence of symptoms (*P=*0.005) and microscopic findings (*P=*0.0003) related to collapsing glomerulopathy in dialysis-dependent group was noted with increased management (*P=*0.01) used in this group for coronavirus disease-2019 infection.

**Conclusion:**

The findings of this study portray the prognostic value of the variables (symptoms and microscopic findings, etc.) reported in the analysis. Hence this study serves as a foundation for future investigations that minimize the study’s limitations to provide a more robust conclusion.

HIGHLIGHTS

- With a mean age of 54.2 years old, respiratory symptoms (59.6%, 95% CI: 50.4–68.2%) were commonly observed.
- Presence of symptoms (P=0.005) was higher in dialysis dependent group.
- Increased management (P=0.01) was observed in dialysis dependent group with coronavirus disease-2019.

## Introduction

Severe acute respiratory syndrome coronavirus 2 (SARS-COV-2) emerged in Wuhan, China, around December 2019 and since then has remained an illness that has devastated the world in all sorts of ways possible. In February 2020, WHO termed the outbreak of the pandemic coronavirus disease-2019 (COVID-19)^1^. Coronavirus typically manifests as acute respiratory distress syndrome, predominantly affecting the respiratory system; most patients are asymptomatic or exhibit mild symptoms^2^. While the target organ is the lung, studies have shown multiple-organ involvement associated with COVID-19 as direct or indirect infection, which may result in death^3^.

Evidence suggests that, among other organs, COVID-19 contributes to adverse renal manifestations such as acute kidney injury (AKI), which is in turn associated with severe mortality and morbidity; the kidneys are involved in a spectrum from a subtle rise in serum creatinine to end-stage failure that may result in death^4^. The literature proposed that AKI results mainly from two direct mechanisms: one is the cytokine storm seen with sepsis, rhabdomyolysis, shock, and hypoxia, while another tool for renal parenchyma damage is increased expression of ACE-2 receptors in renal tubules that allow entry of the SARS-COV-2 virus in cells. Employing the ACE-2 pathway, the SARS-COV-2 virus enters and causes many harmful effects: acute tubular necrosis (ATN), podocyte localization, mitochondrial impairment with ADP/ADP depletion, endothelial damage, and collapsing glomerulopathy (CG)^5^.

COVID-19-associated AKI has been evaluated in studies by clinical and renal biopsy findings. An American multicenter retrospective cohort study, including data from the USA, Switzerland, and India, done in 2021 with the highest number of renal biopsies to date (273), has shown in its native reports that African American patients with COVID-19 have high-risk apolipoprotein genes (APOL1), causing CG amongst the majority (25.8%)^6^. CG is a rare variation of focal segmental glomerulosclerosis that can lead to end-stage renal disease (ESRD). Before COVID-19, many types of viruses, especially HIV outbreaks, had shown expansion in the occurrence of CG as the HIV-1 gene is known for its autoimmune-mediated podocyte damage leading to renal parenchymal injury, an entity named HIVAN (HIV-associated nephropathy). Histopathological findings show podocyte damage, glomerular sclerosis, and a characteristic capillary loop seen to be collapsed^7^.

Given the sporadicity of the data regarding COVID-19, nephrological manifestations are not clearly defined. Despite the progressively advancing knowledge regarding the clinical course of the disease, no effective therapeutic agent has been discovered. Further, research on the clinical course of the disease could prove instrumental in developing an effective therapeutic strategy and understanding the underlying reciprocity between the nephrological manifestations and coronavirus.

As such, through our systematic review and meta-analysis of published literature, we sought to address the questions: (i) What are the clinical and laboratory features of CG associated with COVID-19? (ii) What treatment/management modalities are required for CG in COVID-19? (iii) What are the outcomes of patients with CG secondary to COVID-19? (iv) What are the risk factors and prevalence associated with CG in COVID-19?

## Methods

A review was conducted and reported following the Preferred Reporting Items for Systematic Reviews and Meta-Analyses (PRISMA) 2020 guidelines and criteria^8^, Supplemental Digital Content 2, http://links.lww.com/MS9/A16. PROSPERO was used to register the review protocol (CRD42022302909)^9^. The AMSTAR-2, Supplemental Digital Content 1, http://links.lww.com/MS9/A15 checklist was also utilized to assess this study^10^. Thus the overall confidence in results was rated high.

### Search methods

An extensive literature review was conducted on major databases: PubMed, Google Scholar, China National Knowledge Infrastructure (CNKI) Database, Cochrane Library, and Science Direct. Additionally, MedRxiv and BioRxiv were searched for preprints, and manual searches of leading medical journals were also conducted, covering a timeline of 1 January 2020 to 5 February 2022. Relevant articles were screened from the bibliography of the retrieved articles. The following keywords were used to search: COVID-19 OR SARS-CoV-2, Collapsing Glomerulopathy, Glomerulosclerosis, Focal Segmental, as shown in Supplementary Table 1, Supplemental Digital Content 3, http://links.lww.com/MS9/A17. There were no language limitations. More research was found by scanning key reference lists.

### Selection strategy

Observational studies (cohorts, case series, and case reports) reporting laboratory‐confirmed COVID-19 (real time-PCR), as well as a confirmed diagnosis of CG on kidney biopsy, were considered for inclusion. The study did not include review papers, editorials, and letters that did not contain any original data. A third independent author handled any disputes in selection.

### Data extraction and analysis

Two reviewers, separately and in duplicate, extracted the shortlisted papers into a structured data form. The following data was taken from each study: authors’ names, publication date, country, study setting, type of study, number of patients and age group, patient information (age, gender, and comorbidities), clinical evaluation, radiographic findings, therapeutic regimen (medications), outcomes for both groups (Full recovery, Partial Recovery, Dialysis Dependent, Discharge, and Death).

Comprehensive meta-analysis 3.3.070 software used mean proportions to summarize the categorical data. While the random-effects model in Review Manager 5.4 was used to calculate the RR between the comparison groups during treatment in dialysis-dependent and nondependent. Figures were made using Review Manager 5.4. A *P*-value less than 0.05 was considered statistically significant.

### Risk of bias assessment

A quality assessment tool developed by the National Institute of Health (NIH) for assessing case series was utilized^11^. Two authors assessed the quality of included articles independently. A third author resolved disagreements. Case reports and case series were scored out of 8 and categorized as good quality (score 6–8), fair quality (score 4–5), and poor quality (score <4).

## Results

### Study selection and risk of bias

Upon duplicate removal, 176 studies were found eligible for the title and abstract screening. In all, 130 studies were assessed for full-text eligibility, out of which 38 were eventually included in this review, all of which were observational^12^–^49^. The risk of bias for the studies is depicted in Supplement Table 2, Supplemental Digital Content 3, http://links.lww.com/MS9/A17.

### Patient demographics

Out of 114 patients, 74 (64.9%) patients were males. The mean age of the study population was 54.2 years old, with the youngest being 21 years old. The African Black population (72.8%, 95% CI: 63.9–80.2%) was the commonest amongst the reported ethnicities. Hypertension was present in 77.2% (95% CI: 68.6–84.0%) patients while diabetes was reported in 33.3% (95% CI: 25.3–42.5%) of the cases. Chronic kidney disease (CKD) accounted for the least reported comorbidity at 21.9% (95% CI: 15.3–30.4%). The presence of any autoimmune disease, neoplasm, and obesity was noted in 3.5% (95% CI: 1.3–9.0%), 2.6% (95% CI: 0.9–7.8%), and 21.9% (95% CI: 15.3–30.4%) of the cases, respectively. Overall, around 45.6% (95% CI: 36.7–54.8%) of the patients reported more than one comorbidity.

**Figure 1:**
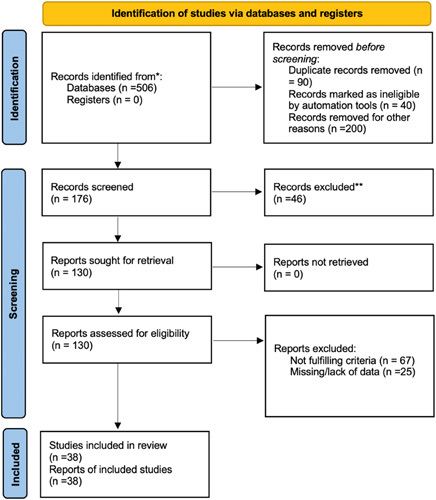
Preferred Reporting Items for Systematic Reviews and Meta-Analyses (PRISMA) diagram of included studies, *n*=38.

**Figure 2:**
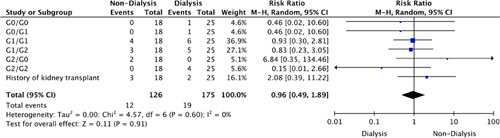
Risk ratio of genetic predisposition.

Our data indicated that 15 (13.2%) of the patients were categorized as being kidney transplant recipients. Furthermore, the G1/G2 ratio was indicated in 14.0% (95% CI: 8.8–21.7%) of the patients, G0/G0 ratio in 1.8% (95% CI: 0.4–6.7%) patients, G1/G1 ratio in 21.1% (95% CI: 14.5–29.5%) patients and G2/G0 ratio in 1.8% (95% CI: 0.4–6.7%) of the patients. Lastly, Figures 1 and 2 shows the genetic predisposition of patients undergoing dialysis-dependent versus nondialysis-dependent management. No statistically significant findings were indicated.

### Clinical symptoms

Table 1 summarizes pooled incidences of clinical symptoms. The most common symptom reported related to COVID-19 infection was in the respiratory system in 59.6% (95% CI: 50.4–68.2%), followed by fever (46.5%, 95% CI: 37.6–55.7%), whereas hematuria (34.2%, 95% CI: 26.1–43.4) was the most observed symptom for CG.

**Table 1:** Clinical symptoms in the included population.

Variables	Frequency (*N*=114)	%	95% CI
Clinical symptoms of COVID-19			
Respiratory distress	20	17.5	11.6–25.6
Respiratory symptoms	68	59.6	50.4–68.2
Gastrointestinal symptoms	29	25.4	18.3–34.2
Fever	52	45.6	36.7–54.8
Fatigue	17	14.9	9.5–22.7
Myalgia	10	8.8	4.8–15.5
Lower extremity edema	19	16.7	10.9–24.7
Clinical symptoms of collapsing glomerulopathy			
Oliguria	18	15.8	10.2–23.7
Flank pain	2	1.8	0.4–6.7
Hematuria	39	34.2	26.1–43.4

COVID-19 indicates, coronavirus disease-2019.

**Figure 3:**
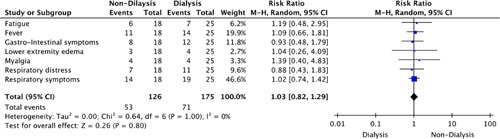
Risk ratio of clinical symptoms for coronavirus disease-2019 (COVID-19) infection.

Figure 3 highlights the RR of reporting any of the symptoms in our comparison groups for COVID-19 infection. This analysis yielded no statistically significant conclusion.

**Figure 4:**
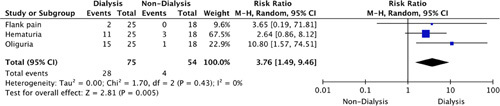
Risk ratio of clinical symptoms for collapsing glomerulopathy.

Furthermore, Figure 4 highlights the RR of reporting any symptoms related to CG. Oliguria (RR: 10.80, 95% CI: 1.57–74.51) was present in dialysis-dependent patients compared to nondialysis-dependent patients alongside an overall increased risk of symptomatic presentation in the dialysis-dependent population (RR: 3.76, 95% CI: 1.49–9.46, *P=*0.005).

### Laboratory and microscopic findings

Table 2 summarize pooled incidences of laboratory and microscopic findings. Proteinuria and raised serum creatinine were the most reported laboratory findings at 89.5% (95% CI: 82.4–93.9%) and 86.8% (95% CI: 79.3–91.9). Acute tubular injury (ATI) (77.2%, 95% CI: 68.6–84.0%) was the most reported microscopic finding amongst the included study population, as shown in Table 2.

**Table 2:** Laboratory and microscopic findings in the included population.

Variables	Frequency (*N*=114)	%	95% CI
Laboratory findings			
Proteinuria	102	89.5	82.4–93.9
Decreased eGFR	14	12.3	7.4–19.7
Nephrotic range proteinuria	78	68.4	59.3–76.3
Raised serum BUN	17	14.9	9.5–22.7
Raised serum creatinine	99	86.8	79.3–91.9
Hypoalbuminemia	58	50.9	41.8–59.9
Raised CRP	23	20.2	13.8–28.5
Raised D-dimer	13	11.4	6.7–18.7
Raised serum ferritin	21	18.4	12.3–26.6
Raised serum cytokines	14	12.3	7.4–19.7
Anemia	13	11.4	6.7–18.7
Pyuria	9	7.9	4.2–14.5
Lymphopenia	9	7.9	4.2–14.5
Reduced sodium urinary excretion	3	2.6	0.9–7.8
Low C3 complement level	4	3.5	1.3–9.0
Microscopic findings			
Tubular atrophy	75	65.8	56.6–73.9
Global capillary collapse	18	15.8	10.2–23.7
Segmental capillary collapse	26	22.8	16.0–31.4
ATI	88	77.2	68.6–84.0
ATN	24	21.1	14.5–29.5
Diffuse foot process effacement	68	59.6	50.4–68.2
Tubuloreticular inclusions	28	24.6	17.5–33.3
Interstitial fibrosis	82	71.9	63.0–79.4
Protein resorption droplets	32	28.1	20.6–37.0
Viral inclusion bodies in the podocyte cytoplasm	4	3.5	1.3–9.0
Renal epithelial cells	9	7.9	4.2–14.5

ATI indicates, acute tubular injury; ATN, acute tubular necrosis; BUN, blood urea nitrogen; CRP, C-reactive protein; eGFR, estimated glomerular filtration rate.

**Figure 5:**
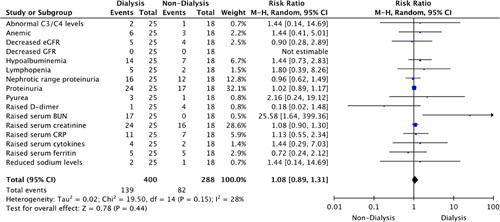
Risk ratio of laboratory findings. BUN, blood urea nitrogen; CRP, C-reactive protein; eGFR, estimated glomerular filtration rate.

**Figure 6:**
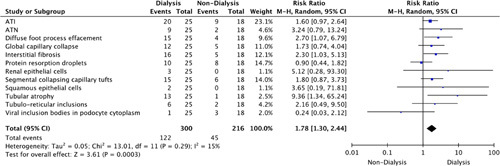
Risk ratio of microscopic findings. ATI, acute tubular injury; ATN, acute tubular necrosis.

Risk ratio analysis indicated that during treatment, dialysis-dependent patients were at an increased risk of reporting raised serum blood urea nitrogen (RR: 25.58, 95% CI: 1.64–399.36) and tubular atrophy (RR: 9.36, 95% CI: 1.34–65.24) compared to nondialysis-dependent patients as indicated in Figures 5 and 6, respectively. In addition, a statistically significant microscopic finding in dialysis-dependent patients (RR: 1.78, 95% CI: 1.30–2.44, *P=*0.0003) was observed (Fig. 6).

### Managements and outcomes

Clinical management was reported in Table 3. Antibiotics (21.9%, 95% CI: 15.3–30.4%) was the commonest treatment regimen employed for COVID-19, whereas dialysis (57.0%, 95% CI: 47.8–65.8%) was the mainstay management for CG.

**Table 3:** Management and outcomes in the included population.

Variables	Frequency (*N*=114)	%	95% CI
Management for COVID-19			
Steroids	24	21.1	14.5–29.5
Hydroxychloroquine	16	14.0	8.8–21.7
Antibiotics	25	21.9	15.3–30.4
Remdesivir	5	4.4	1.8–10.1
Protease inhibitor	1	0.9	0.1–6.0
Plasma exchange	1	0.9	0.1–6.0
Oseltamivir	2	1.8	0.4–6.7
Oxygen supplementation	19	16.7	10.9–24.7
Management for collapsing glomerulopathy			
Dialysis	65	57.0	47.8–65.8
Steroids	24	21.1	14.5–29.5
CRRT	3	1.8	0.4–6.7
Antibiotics	8	7.0	3.5–13.4
Outcomes			
Hospitalized/under observation	33	28.9	21.4–37.9
No improvement in kidney function	19	16.7	10.9–24.7
Partial improvement in kidney function	37	32.5	24.5–41.6
Full recovery	11	9.6	5.4–16.6
Dialysis dependent	30	26.3	19.1–35.1
Discharged	56	49.1	40.1–58.2
Death	8	7.0	3.5–13.4

COVID-19 indicates, coronavirus disease-2019; CRRT, continuous renal replacement therapy.

**Figure 7:**
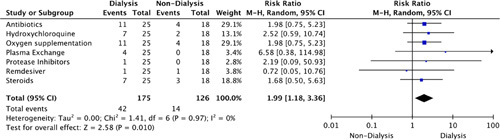
Risk ratio for clinical management of coronavirus disease-2019 (COVID-19) infection.

**Figure 8:**
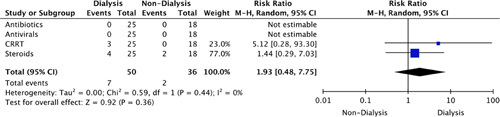
Risk ratio of clinical management of collapsing glomerulopathy. CRRT, continuous renal replacement therapy.

An overall increased management was observed for COVID-19 treatment (RR: 1.99, 95% CI: 1.18–3.36, *P=*0.01) and CG (RR: 1.93, 95% CI: 0.48–7.75, *P=*0.36) in dialysis-dependent patients (Figs 7, 8).

**Figure 9:**
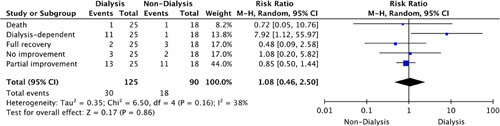
Risk ratio of outcomes.

A total of 26.3% (95% CI: 19.1–35.1%) of the patients were eventually categorized as dialysis dependent upon the completion of their treatment. Full recovery was noted in 9.6% (95% CI: 5.4–16.6%) of the patients with eight deaths (7.0%, 95% CI: 3.5–13.4%) reported (Table 3). Figure 9 highlights the outcomes as indicated in the comparison groups.

## Discussion

Among 38 observational studies, we have received demographic information, laboratory and histopathological findings, clinical symptoms, and management and outcome reports for 144 patients. Most studies have shown the incidence of kidney disease in Black Africans and African Americans above other ethnicities due to the homozygous APOL1 – G1 and G2 genes, as G1/G1 is seen highest amongst our patient’s group (21.1%). The presence of APOL1 is known to directly cause CG, nephrotic syndrome, AKI, and ESRD^35^–^50^. Incidence is seen higher in males (62.7%); however, some studies report that prevalence is equal amongst genders and that females in lower-income countries are less likely to be admitted to hospitals due to the inconvenience of health care provision^51^–^56^. Studies also state that females have a higher risk of more severe complications due to these shortcomings^57^.

Among our patient pool, hypertension was the most associated comorbidity (77.2%), followed by diabetes mellitus (DM) in (33.3%) of patients. Studies state DM is the leading cause of hypertension^58^. Comorbidities like hypertension, DM, and obesity are seen to develop in COVID-19-positive patients due to factors including older age, oxidative stress, genetics, and medication. Further progression of DM and hypertension in COVID-19 is seen to result in ESRD^59^–^61^.

The majority of patients in our pooled analysis presented with respiratory symptoms (59.6%) followed by fever (45.6%); these symptoms are classically present as indicators of COVID-19 and SARS-COV-2^62^. Before COVID-19, there have been cases where CG secondary to pulmonary tuberculosis was diagnosed, caused by the expression of multiple viruses such as HIV, Epstein–Barr virus, and parvovirus^63^,^64^.

CG is a variation of focal segmental glomerulosclerosis known to progress to ESRD. As our results state increased incidence in proteinuria (89.5%), raised serum creatinine (86.8%), and hypoalbuminemia (50.9%), It is seen to be caused most likely due to ACE-2 expression in proximal convoluted tubules (as it occurs in multiple other organs) as COVID-19 infection in cells disrupt renal parenchyma leading to ATI/ATN. ATI is a commonly reported histopathological finding in our study (77.2%); it is one of the complications seen in AKI, COVID-19-associated nephropathy, and CG^5^,^17^,^23^,^24^,^65^. Histological microscopic findings have shown diffuse podocyte foot process effacement; this specified podocytopathy is commonly seen in CG presenting with proteinuria. Dysregulation of podocytes would consequently play a role in decreasing glomerular filtration rate due to renal damage^5^,^36^,^66^–^68^.

Data gathered from dialysis-dependent and independent patients show that initially, both groups had equal RRs; symptoms like coughing were seen to be more common in dialysis-dependent patients. The higher incidence of CG secondary to pulmonary tuberculosis alongside proteinuria in dialysis-dependent patients explains more coughing, as some studies have reported^36^,^64^,^67^–^69^. Furthermore, on treatment with antibiotics in most patients (21.9%), nondialysis-dependent patients had improvement from the medications far better than dialysis-dependent patients. On treatment, dialysis-dependent patients seemed to have an increased risk for diffused foot process effacement and deranged laboratory results; this is explained by a study as dialysis-dependent patients are more prone to drug toxicity than nondialysis-dependent patients on the same amount of medication. These drugs include a few opioids like codeine. Morphine and oxycodone, many antimicrobials like quinolones, sulfamethoxazole with trimethoprim, glycopeptides, and aminoglycosides, alongside certain anticoagulants such as warfarin, heparin, dabigatran, and rivaroxaban, for dialysis-dependent patients with diabetes insulin and insulin secretagogues all require a dose adjustment and reduction for dialysis-dependent patients^70^. We recommend further research to be conducted on the limiting doses of COVID-19-positive dialysis and nondialysis-dependent patient medication for a lower RR; the use of antiviral drugs, including acyclovir and its prodrugs, famciclovir, and valaciclovir can be keenly studied as to which levels they are tolerable by the dialysis-dependent patients and able to combat COVID-19.

Our analysis showed that there was a significantly increased risk of developing collapsing capillary tufts, epithelial hyperplasia, epithelial hypertrophy, hematuria, interstitial fibrosis, presence of protein resorption droplets, pyuria, tubular atrophy, and viral inclusion bodies in podocyte cytoplasm; such a poor prognosis has been reported in the scientific literature^71^–^73^. Khan *et al*.^7^, who conducted a cohort study with a longitudinal follow-up for 10 years or sample expiration date of 10 years with sample size *n*=621, reported microscopic hematuria as a predictor of CKD, with such patients being twice more likely to progress toward ESRD. This is supported by Moreno *et al*.^74^, who indicated in their study that hematuria led to tubular injury, as shown by our analysis, which subsequently caused kidney damage and increased the occurrence of ESRD. Therefore, the findings of our study related to microscopic histopathological abnormalities, supported by the literature, are crucial in predicting the prognosis of COVID-19 patients who encountered CG.

Full recovery was only observed in 9.6% of patients, while 16.7% showed no improvement in kidney function. We observed that dialysis-dependent patients have a slightly higher death rate of 0.65 times more than nondialysis-dependent patients due to comparatively severe kidney damage and a lower chance of recovery. The total number of dialysis-dependent patients (26.3%) had shown partial recovery. We know that patients with underlying kidney disease such as CKD and renal transplantation are at risk of renal failure and dialysis dependency after exposure to COVID-19 infection; this further supports the association of kidney damage and particularly CG during the onset of COVID-19^35^,^75^–^77^.

The results indicating a positive correlation between nephrological disruption and COVID-19, indeed strengthen the value of this study. While there have been previous literature covering the associations of renal diseases with respiratory diseases, however this study focuses on additional factors such as global epidemiology, prevalence, management, treatment, complications, and outcomes of sample studies. In addition, the detailed comparison and analysis of the correlation between CG and COVID-19 has been a priority in this study.

This review has several limitations. The key restriction is the fast-changing data and the difficulty of keeping up with the disease process’s rapid participation and publication rate. Another significant limitation is that many patients did not go through renal biopsies due to various clinical and laboratory parameters that render renal biopsies a relatively contraindicated procedure; these were sick patients on ventilator support, and thrombocytopenia to name a few. Nevertheless, the authors did their best to keep the search data up to date. Still, it is probable that by the time the review reaches the public, numerous new therapy and management options will have emerged, necessitating ongoing research and updates.

Furthermore, most of the studies hail from high-income countries (*n*=37, 97%). As a result, findings from these studies may apply to the socioeconomic and health development status of other high-income countries, but not to low-income ones. Other researchers could improve future studies by incorporating a larger sample size and including studies from low-income and middle-income countries to provide results that can be generalized globally.

Consequently, the article implies collective data on a global scale associating CG and COVID-19 can provide information in a concise manner. Future studies can gain data accumulated in a single source manner for the nephrological associations with COVID-19. This will ensure further expansion and insight in researches in the field of nephrology postpandemic.

## Conclusion

In this research, we attempted to collect all studies that reported cases of CG to quantify them properly. Our study highlights the key correlation between the presence of symptoms, microscopic findings, and increased management of COVID-19 in the dialysis-dependent treatment group. These observations hold significance as being potentially used for prognostic purposes in the near future. While we strived to be holistic in our approach, further research is required to comprehend the pathophysiology of CG in the COVID-19 infection fully.

## Ethical approval

Since it is a systematic review thus ethical approval is not required.

## Patient consent

Not applicable.

## Sources of funding

None.

## Author contribution

M.A.Q., S.S.T., A.A., and M.R.A.R. conceptualized the study. L.M.K., J.N., and M.A.Q. reviewed the literature. A.A., K.Q., and L.M.K. contributed to data extraction. M.A.Q. and A.Y. contributed toward the data interpretation and analysis. All authors contributed toward the write up and approved the final version of the manuscript.

## Conflicts of interest disclosure

There are no conflicts of interest.

## Research registration unique identifying number (UIN)

1. Name of the registry: International Prospective Register of Systematic Review (PROSPERO).

2. The unique identifying number or registration ID: CRD42022302909.

3. Hyperlink to your specific registration: https://www.crd.york.ac.uk/prospero/display_record.php?ID=CRD42022302909

## Guarantor

M.A. Qamar.

## Provenance and peer review

Not commissioned, externally peer reviewed.

## Availability of data

All data is present in the main manuscript and supplemental file.

## Supplementary Material
