# Supplementary material for: Collapsing focal segmental glomerulosclerosis secondary to COVID-19: A systematic review and meta-analysis
Source: Ann Med Surg (Lond). 2023 Feb 7;85(2):92–101. doi: 10.1097/MS9.0000000000000107 (PMC9949810; doi:10.1097/MS9.0000000000000107)
Supplement: Supplementary file 3 [file ms9-85-092-s003.docx]

**Table 1. Search strategy used for included databases**

| **Database/ Register** | **Search Strategy** |
| --- | --- |
| **PubMed** | ((("collapsed"[All Fields] OR "collapses"[All Fields] OR "collapsibility"[All Fields] OR "collapsible"[All Fields] OR "collapsing"[All Fields] OR "shock"[MeSH Terms] OR "shock"[All Fields] OR "collapse"[All Fields]) AND ("glomerulopathies"[All Fields] OR "glomerulopathy"[All Fields])) OR (("collapsed"[All Fields] OR "collapses"[All Fields] OR "collapsibility"[All Fields] OR "collapsible"[All Fields] OR "collapsing"[All Fields] OR "shock"[MeSH Terms] OR "shock"[All Fields] OR "collapse"[All Fields]) AND ("glomerulosclerosis, focal segmental"[MeSH Terms] OR ("glomerulosclerosis"[All Fields] AND "focal"[All Fields] AND "segmental"[All Fields]) OR "focal segmental glomerulosclerosis"[All Fields] OR ("focal"[All Fields] AND "segmental"[All Fields] AND "glomerulosclerosis"[All Fields]))) OR ("glomerulosclerosis, focal segmental"[MeSH Terms] OR ("glomerulosclerosis"[All Fields] AND "focal"[All Fields] AND "segmental"[All Fields]) OR "focal segmental glomerulosclerosis"[All Fields] OR ("focal"[All Fields] AND "segmental"[All Fields] AND "glomerulosclerosis"[All Fields])) OR "glomerulosclerosis"[All Fields] OR ("nephrotic syndrome"[MeSH Terms] OR ("nephrotic"[All Fields] AND "syndrome"[All Fields]) OR "nephrotic syndrome"[All Fields]) OR ("kidney diseases"[MeSH Terms] OR ("kidney"[All Fields] AND "diseases"[All Fields]) OR "kidney diseases"[All Fields] OR "nephropathies"[All Fields] OR "nephropathy"[All Fields]) OR ("podocytopathies"[All Fields] OR "podocytopathy"[All Fields]) OR ("podocytopathies"[All Fields] OR "podocytopathy"[All Fields]) OR (("acute"[All Fields] OR "acutely"[All Fields] OR "acutes"[All Fields]) AND ("kidney diseases"[MeSH Terms] OR ("kidney"[All Fields] AND "diseases"[All Fields]) OR "kidney diseases"[All Fields] OR ("kidney"[All Fields] AND "disease"[All Fields]) OR "kidney disease"[All Fields])) OR ("acute kidney injury"[MeSH Terms] OR ("acute"[All Fields] AND "kidney"[All Fields] AND "injury"[All Fields]) OR "acute kidney injury"[All Fields]) OR ("renal insufficiency"[MeSH Terms] OR ("renal"[All Fields] AND "insufficiency"[All Fields]) OR "renal insufficiency"[All Fields] OR ("renal"[All Fields] AND "failure"[All Fields]) OR "renal failure"[All Fields]) OR ("kidney failure, chronic"[MeSH Terms] OR ("kidney"[All Fields] AND "failure"[All Fields] AND "chronic"[All Fields]) OR "chronic kidney failure"[All Fields] OR ("end"[All Fields] AND "stage"[All Fields] AND "renal"[All Fields] AND "disease"[All Fields]) OR "end stage renal disease"[All Fields]) OR ("kidney failure, chronic"[MeSH Terms] OR ("kidney"[All Fields] AND "failure"[All Fields] AND "chronic"[All Fields]) OR "chronic kidney failure"[All Fields] OR "esrd"[All Fields]) OR "FSGS"[All Fields] OR "cFSGS"[All Fields] OR ("comput geosci"[Journal] OR "cg"[All Fields]) OR "AKI"[All Fields]) AND ("covid 19"[All Fields] OR "covid 19"[MeSH Terms] OR "covid 19 vaccines"[All Fields] OR "covid 19 vaccines"[MeSH Terms] OR "covid 19 serotherapy"[All Fields] OR "covid 19 serotherapy"[Supplementary Concept] OR "covid 19 nucleic acid testing"[All Fields] OR "covid 19 nucleic acid testing"[MeSH Terms] OR "covid 19 serological testing"[All Fields] OR "covid 19 serological testing"[MeSH Terms] OR "covid 19 testing"[All Fields] OR "covid 19 testing"[MeSH Terms] OR "sars cov 2"[All Fields] OR "sars cov 2"[MeSH Terms] OR "severe acute respiratory syndrome coronavirus 2"[All Fields] OR "ncov"[All Fields] OR "2019 ncov"[All Fields] OR (("coronavirus"[MeSH Terms] OR "coronavirus"[All Fields] OR "cov"[All Fields]) AND 2019/11/01:3000/12/31[Date - Publication]) OR ("coronavirus"[MeSH Terms] OR "coronavirus"[All Fields] OR "coronaviruses"[All Fields]) OR ("sars cov 2"[MeSH Terms] OR "sars cov 2"[All Fields] OR "severe acute respiratory syndrome coronavirus 2"[All Fields]) OR ("sars cov 2"[MeSH Terms] OR "sars cov 2"[All Fields] OR "sars cov 2"[All Fields])) |
| - **Google Scholar** - **China National Knowledge Infrastructure (CNKI)** - **Cochrane Library** - **Science Direct** - **MedRxiv** - **BioRxiv** | (COVID-19 OR SARS-CoV-2) AND (Collapsing Glomerulopathy OR Glomerulosclerosis OR Focal Segmental) |

Table 2A: Quality assessment of included studies

| # | First  author | Was the study question or objective clearly stated? | Was the study population clearly and fully described, including a case definition? | Were the cases consecutive? | Were the subjects comparable? | Was the intervention clearly described? | Were the outcome measures clearly defined, valid, reliable, and implemented consistently across all study participants? | Was the length of follow-up adequate? | Were the statistical methods well described? | Were the results well described? | Total | Level of Evidence | |
| --- | --- | --- | --- | --- | --- | --- | --- | --- | --- | --- | --- | --- | --- |
| 1 | Sebastien Kissling et al. | yes | yes | NA | NA | Yes | Yes | Yes | NA | N/A | 5 | fair |  |
| 2 | Christopher P. Larsen et al. | yes | Yes | NA | NA | Yes | Yes | No | NA | N/A | 4 | Fair |  |
| 3 | Sarika Deshmukh et al. | yes | Yes | N/A | N/A | Yes | Yes | Yes | N/A | N/A | 5 | fair |  |
| 4 | Kristin Meliambro et al. | Yes | yes | N/A | N/A | yes | yes | yes | N/A | N/A | 5 | fair |  |
| 5 | Hassan Izzedine et al. | yes | No | Yes | Yes | Yes | Yes | Yes | NA | N/A | 6 | good |  |
| 6 | Rebacca Noble et al. | yes | No | Yes | Yes | Yes | Yes | Yes | NA | N/A | 6 | good |  |
| 7 | Yonatan Peleg et al. | Yes | Yes | N/A | N/A | Yes | Yes | Yes | N/A | N/A | 5 | fair |  |
| 8 | G. J. Hoilat et al. | Yes | No | NA | NA | Yes | Yes | Yes | NA | N/A | 4 | fair |  |
| 9 | Helene Lazareth et al. | Yes | Yes | NA | NA | Yes | Yes | Yes | NA | N/A | 5 | Fair |  |
| 10 | Sandeep Magoon et al. | Yes | Yes | Yes | Yes | Yes | Yes | Yes | NA | N/A | 7 | Good |  |
| 11 | Yannick M Nlandu | Yes | No | NA | NA | Yes | Yes | Yes | NA | N/A | 4 | Fair |  |
| 12 | Francois Gaillard et al. | Yes | Yes | NA | NA | Yes | Yes | No | NA | N/A | 4 | Fair |  |
| 13 | Onoriode Kesiena et al. | yes | Yes | NA | NA | Yes | Yes | No | NA | N/A | 4 | Fair |  |
| 14 | Sasmit Roy et al. | Yes | yes | N/A | N/A | yes | yes | yes | N/A | N/A | 5 | fair |  |
| 15 | Yuvraj Sharma et al. | Yes | Yes | N/A | N/A | Yes | Yes | No | N/A | N/A | 4 | Fair |  |
| 16 | Timothee Laboux et al. | Yes | Yes | Yes | Yes | Yes | Yes | Yes | N/A | N/A | 7 | Good |  |
| 17 | Rajib K. Gupta, | Yes | yes | yes | yes | yes | yes | no | N/A | N/A | 6 | good |  |
| 18 | Varun Malhotra, MD et al. | Yes | Yes | N/A | N/A | Yes | Yes | Yes | N/A | N/A | 5 | fair |  |
| 19 | Bernard S. Kadosh et al. | Yes | Yes | N/A | N/A | Yes | Yes | Yes | N/A | N/A | 5 | fair |  |
| 20 | Tyler Tancredi et al. | Yes | yes | N/A | N/A | yes | yes | yes | N/A | N/A | 5 | fair |  |
| 21 | Christophe Masset et al. | Yes | Yes | N/A | N/A | Yes | Yes | Yes | N/A | N/A | 5 | fair |  |
| 22 | Shirui Chen et al | yes | yes | N/A | N/A | yes | yes | no | N/A | N/A | 4 | fair |  |
| 23 | Janewit Wongboonsin et al | yes | yes | N/A | N/A | yes | yes | yes | N/A | N/A | 5 | fair |  |
| 24 | Aymeric couturier et al | yes | yes | yes | yes | yes | yes | no | N/A | N/A | 6 | good |  |
| 25 | Emily Daniel et al | yes | yes | yes | yes | yes | yes | yes | yes | no | 8 | good |  |
| 26 | Purva Sharma et al | yes | yes | yes | yes | yes | yes | no | no | N/A | 6 | good |  |
| 27 | Aneesha A. Shetty et al | yes | yes | yes | yes | yes | yes | yes | no | no | 7 | Good |  |
| 28 | Is-haq O Malik et al | yes | yes | N/A | N/A | yes | yes | yes | N/A | N/A | 5 | fair |  |
| 29 | Shreeram Akilesh et al | yes | yes | yes | yes | yes | yes | yes | no | no | 7 | good |  |
| 30 | Huijuan Wu et al. | Yes | Yes | Yes | Yes | Yes | Yes | Yes | NA | N/A | 7 | Good |  |
| 31 | Samih H. Nasr et al | yes | yes | yes | yes | yes | yes | yes | no | no | 7 | good |  |
| 32 | Samy Hakroush et al | yes | yes | N/A | N/A | yes | yes | no | N/A | N/A | 4 | fair |  |
| 33 | Mathilde Dargelos et al | yes | no | yes | yes | yes | yes | no | N/A | N/A | 5 | fair |  |

Table 2B: Quality assessment of included studies

| s. no | Study name | Was the research question or objective in this paper clearly stated? | Was the study population clearly specified and defined? | Was the participation rate of eligible persons at least 50%? | Were all the subjects selected or recruited from the same or similar populations (including the same time period)? Were inclusion and exclusion criteria for being in the study prespecified and applied uniformly to all participants? | Was a sample size justification, power description, or variance and effect estimates provided? | For the analyses in this paper, were the exposure(s) of interest measured prior to the outcome(s) being measured? | Was the timeframe sufficient so that one could reasonably expect to see an association between exposure and outcome if it existed? | For exposures that can vary in amount or level, did the study examine different levels of the exposure as related to the outcome (e.g., categories of exposure, or exposure measured as continuous variable)? | Were the exposure measures (independent variables) clearly defined, valid, reliable, and implemented consistently across all study participants? | Was the exposure(s) assessed more than once over time? | Were the outcome measures (dependent variables) clearly defined, valid, reliable, and implemented consistently across all study participants? | Were the outcome assessors blinded to the exposure status of participants? | Was loss to follow-up after baseline 20% or less? | Were key potential confounding variables measured and adjusted statistically for their impact on the relationship between exposure(s) and outcome(s)? | total |
| --- | --- | --- | --- | --- | --- | --- | --- | --- | --- | --- | --- | --- | --- | --- | --- | --- |
| 34 | Satoru Kudose et al | yes | yes | N/A | no | yes | yes | N/A | No | yes | No | yes | N/A | yes | yes | 8 |
| 35 | Nikolina Basic-Jukic et al | yes | yes | N/A | yes | Yes | yes | yes | No | yes | No | yes | N/A | yes | no | 9 |
| 36 | Satoru Kudose | yes | yes | N/A | yes | yes | yes | yes | no | yes | no | yes | no | yes | no | 9 |
| 37 | Skylar L. Nahi, et al | yes | yes | N/A | yes | Yes | yes | yes | yes | yes | yes | yes | no | yes | no | 11 |
| 38 | HélènePéré et al | yes | yes | N/A | yes | Yes | yes | yes | no | yes | no | yes | no | yes | no | 9 |
